# Effect of Marine Basidiomycetes *Fulvifomes* sp.-Derived Ergosterol Peroxide on Cytotoxicity and Apoptosis Induction in MCF-7 Cell Line

**DOI:** 10.3390/jof5010016

**Published:** 2019-02-13

**Authors:** Mano Govindharaj, Sathishkumar Arumugam, Grace Nirmala, Mausumi Bharadwaj, Kalaiselvam Murugiyan

**Affiliations:** 1 Centre of Advanced Study in Marine Biology, Annamalai University, Parangipettai 608 502, India; mano.govindraj@gmail.com; 2 Molecular Biology Group, National Institute of Cancer Prevention and Research, Noida 201 301, India; sathiishkumarbiotech@gmail.com (S.A.); mausumi.bharadwaj@gmail.com (M.B.); 3 Experimental Cancer Therapeutics and Chemical Biology Laboratory, UM-DAE Centre for Excellence in Basic Sciences, Kalina, Mumbai 400098, India; gracenirmalaj@gmail.com

**Keywords:** Marine Basidiomycetes, ergosterol peroxide, apoptosis induction, breast cancer

## Abstract

The aim of the present study is to extract the bioactive compounds which can induce the apoptosis in breast cancer cell line MCF-7 by marine basidiomycetes. Internal Transcribed Spacer (ITS) sequences based molecular taxonomic study confirmed that collected the marine basidiomycetes belongs to *Fulvifomes* sp. Further, the isolated compounds from the *Fulvifomes* sp. confirmed as ergosterol peroxide (EP) by spectroscopic studies. The compound inhibited 50% of the cell growth (IC50) at the concentration of 40 µg/mL and induced 90% cell death (IC 90) at the concentration of 80 µg/mL. The ergosterol peroxide generated Reactive Oxygen Species (ROS) and induced apoptotic cell death in MCF-7. Ethidium bromide/Acridine Orange (Et/Br) staining showed the increased number of early and late apoptosis in treated MCF-7 cells. The compounds treated cells indicated the significant loss of mitochondrial membrane potential (Δψm) with *p* < 0.05. The induction of apoptosis by marine basidiomycetes derived ergosterol peroxide was confirmed by chromatin condensation in MCF7 cells using Hoechst staining 33342.

## 1. Introduction

The phylum of Basidiomycetes is a large taxon consisting of more than 30,000 species of fungi. This is a common mushrooms species that includes toadstools, bracket fungi, puffballs, earth balls, earthstars, stinkhorns, false truffles, and jelly fungi. Most basidiomycetes are terrestrial with wind-dispersed spores, but several basidiomycetes grow in marine environments [1]. The secondary metabolites produced by wild basidiomycetes are known for their natural antimicrobial activity and anticancer efficacy; they are also used as antioxidants. Among the pharmaceutical and food industries, basidiomycetes have received considerable attention due to their vast availability of phenolic compounds, polypeptides, terpenes, steroids and lectins, polysaccharides peptides, and polysaccharide–protein complexes [2]. Basidiomycetes have been reported to occur in different terrestrial environments which plays important role in ecological balance of the forests by parasitic and symbiotic relationships [3]. However, little is known regarding marine basidiomycetes, specifically their dynamic and community structure in mangrove environments [4]. In previous studies, the marine basidiomycetes polypore sp. was observed to be associated with mangroves plants [5]. The basidiomycetes *Fomes mangrovicus* was found on mangroves within the Tai National Park in Thailand [6]. However, few studies have examined the distribution and classification of marine basidiomycetes, and no report has examined their bioactive potential. Marine basidiomycetes grow in extreme temperatures, lights, pressures, and diverse saline environments. The adaptation to these harsh and challenging environments has led to rich bioactivity of marine basidiomycetes-derived compounds, which could potentially boost the cytotoxicity against cancer cell proliferation. Breast cancer is the number one cancer among Indian females, with an age-adjusted rate as high as 25.8 per 100,000 women and mortality 12.7 per 100,000 women [7]. In this context, the present investigation attempts to isolate the bioactive compound from marine basidiomycetes to check the anti-breast cancer activity in MCF-7 cell line through apoptosis induction.

## 2. Materials and Methods

### 2.1. Cell Culture

MCF-7 cells were provided by the National Centre for Cell Science (Pune, India) and cultured in RPMI 1640 supplemented medium with 10% Fetal Bovine Serum (FBS), penicillin (100 IU/mL) and streptomycin (100 μg/mL) at 37 °C with 5% of CO_2_ in a humidified atmosphere. For treatment, the marine basidiomycetes-derived Ergosterol Peroxide (EP) was dissolved in the stock solution of Dimethyl Sulfoxide (DMSO) and further diluted with culture medium (without FBS) in different concentrations.

### 2.2. Sample Collection and Identification

During the rainy season, the healthy and young fruiting body of a basidiomycetes mushroom was collected from Muthupet mangrove forest (10.4 °N latitude, 79.5 °E longitude), Tamil Nadu, India. Photographs of the fruit body were taken before and after collection from their habitat. Morphological features of the specimen, including the color of the cap, gills, and mode of attachment were recorded for identification.

### 2.3. Extraction and Purification

The samples were cleaned with distilled water and dried at 50 °C in a hot air oven to remove moisture content; fine powders were obtained by grinding in a mixer grinder. Five hundred grams of powdered sample was mixed with 95% of ethanol for ethanolic extraction and extracted three times at 25 °C for two days in a shaker incubator. The ethanolic extract was centrifuged at 3800 rpm for 45 min and the supernatant was lyophilized. Subsequently, the purification process was conducted via silica gel chromatography (60–120 µm mesh size, 8 cm × 200 cm dimension of the column). The obtained lyophilized extracts were mixed in chloroform and loaded on to the mobile phase of a pre-packed silica gel column. Further, the compounds were fractionated using a solvent gradient system. In total, 28 fractions (1–28) were collected, and the fractions with the same Rf values were mixed together and grouped. Among the collected 28 fractions, Fraction-1 (1–5), Fraction-2 (6–11), Fraction-3 (12–16), Fraction-4 (17–20), Fraction-5 (21–25), and Fraction-6 (26–28) were analyzed for anticancer activity. Among these six fractions, fraction-5 (21–25) showed good anticancer effect. Subsequently, CHCl_3_/CH_3_OH from 0:10 to 2:8 gradients were used for the elution with the flow rate of 1 mL/min and fractions were collected (Fractions 1–10). Fractions were again examined for anticancer activity. The best anticancer activity was absorbed from Fraction-4; it was also subjected to high performance liquid chromatography (HPLC) separation.

### 2.4. Semi-Preparative High Performance Liquid Chromatography (HPLC) Analysis

Separation was achieved by column YMC-pack ODS-A (10 × 250 mm, 5 µM, 4 mL/min) in semi-prep-HPLC. The highly active fraction was subjected to purity assessment by Shimadzu 9A system (C18 column with a UV detector). An injection volume of 20 µL of sample was injected, and the detection wavelength was set at 240 nm. The mobile phase consisted of chloroform/methanol (10:90) and a flow rate of 1.0 mL/min was used.

### 2.5. Fourier-Transform Infared (FTIR) Spectroscopy Analysis

The chemical composition of marine basidiomycete mushroom-derived extract was studied using a FTIR (Thermo Scientific Nicolet-380, Wilmington, DE, USA) spectrophotometer. The extract solution was mixed with KBr pellets and characterized in the range of 4000–5000 cm^−1^.

### 2.6. Nuclear Magnetic Resonance (NMR) Spectroscopy

The bioactive fraction was analyzed according to the ^1^H NMR and ^13^C NMR spectrum to identify the type of compounds in the extracts of the marine basidiomycetes mushroom. Bioactive fraction sample was dissolved in dimethyl sulfoxide d6 (DMSO-d6) and 1% TMS (tetramethylsilane) was used as an internal standard for calibrating chemical shift of ^1^H dissolved in chloroform-d. Subsequently, 0.5 mL of the sample in DMSO was dissolved in 2.5 mL of chloroform-d solvent containing 1% TMS at 15 °C. The solvent chloroform-d provided an integral field frequency clock signal.

### 2.7. MTT Assay

Toxicity of MCF-7 cells was determined by MTT (3-(4,5-dimethylthiazol-2-y)-2,5-diphenyltetrazolium bromide) assay. The cell seeding density of 8 × 104 cells/well were seeded in a 96 well culture plate and incubated at 37 °C with 5% CO_2_. After cells were completely attached, the marine basidiomycetes-derived ergosterol peroxide, which had been dissolved in culture medium in various concentrations, was added to the cells and incubated for 24 h. Subsequently, MTT (0.5 mg/mL) was added to the cells and kept for incubation for 4 h. In each well, 100 μL of DMSO was added to dissolve the formed formazan crystals. Absorbance at 570 nm was measured with an ELISA plate reader. The percentage of cell viability was determined by using following formula, Cell viability = (OD of test samples)/(OD of control samples) × 100. The IC50 value of the compound was determined based on the 50% reduction of cell viability [8].

### 2.8. Assessment of Reactive Oxygen Species (ROS) by DCFH-DA

Measurement of cellular reactive oxygen species (ROS) production was performed by dichlorodihydrofluorescein diacetate assay (DCFH-DA). Briefly, in 24 well plates, the MCF-7 cells were seeded at a density of 2 × 10^5^ cells/ml in each well and incubated for 24 h at 37 °C. After incubation, 1× PBS was used to wash the cells and again incubated with 10 μL of DCFH-DA for 1 h. The inverted microscope Olympus IX-71 (Center Valley, PA, USA) was employed to determine the fluorescence intensity of the DCF [9].

### 2.9. Assessment of Mitochondrial Membrane Potential (MMP)

In a 24 well plate, cells were seeded at a density of 2 × 10^5^ cells/mL in each well and cultured for 24 h at 37 °C. Cells were then treated with marine basidiomycetes-derived ergosterol peroxide and incubated at 37 °C after adding 10 μg/mL of Rhodamine 123 (Rh-123) fluorescent dye. Later, ice-cold PBS was used to wash the cells in the plate and detach them to evaluate the loss of mitochondrial membrane potential with the help of fluorescence microscope images (Olympus IX-71, Center Valley, PA, USA). Finally, the data were generated on the basis of the percentage of cells stained by RH-123 [10].

### 2.10. AO/EtBr Staining

The AO/EtBr double staining test was employed to analyze the morphological changes of apoptosis. Briefly, 2 × 10^5^ cells were seeded in each well of a 24 well plate and incubated for 24 h and then treated with marine basidiomycetes-derived ergosterol peroxide for 24 h at 37 °C. Afterward, cells were washed twice with 1× PBS for further staining with 5 μL of AO/EB. Finally, fluorescence microscopy (Olympus IX-71, USA) was employed to visualize the morphological changes of cells.

### 2.11. Nuclear Damage Observed with Hoechst 33258 Staining

Cells were cultured in 24 well plates, and treated with two varying concentrations of ergosterol peroxide with 40, 80 µg/mL at 37 °C for 24 h. The untreated sample was used as a control. Subsequently, cells were washed with cold 1× PBS and fixed with 3.7% paraformaldehyde at 25 °C. Then, cells were stained using Hoechst 33258 (5 µL/mL in PBS) at 25 °C for 10 min. Lastly, nuclei of the cells were focused by a fluorescent microscope (Olympus IX-71, USA) [11].

### 2.12. Statistical Analysis

All experimental data were represented as mean ± SD. The statistical analysis was done by a one-way ANOVA test between the groups of treated and control. The difference was considered as significant for *p* < 0.05.

## 3. Results

### 3.1. Morphological Characteristics of Marine Basidiomycetes

**Figure 1:**
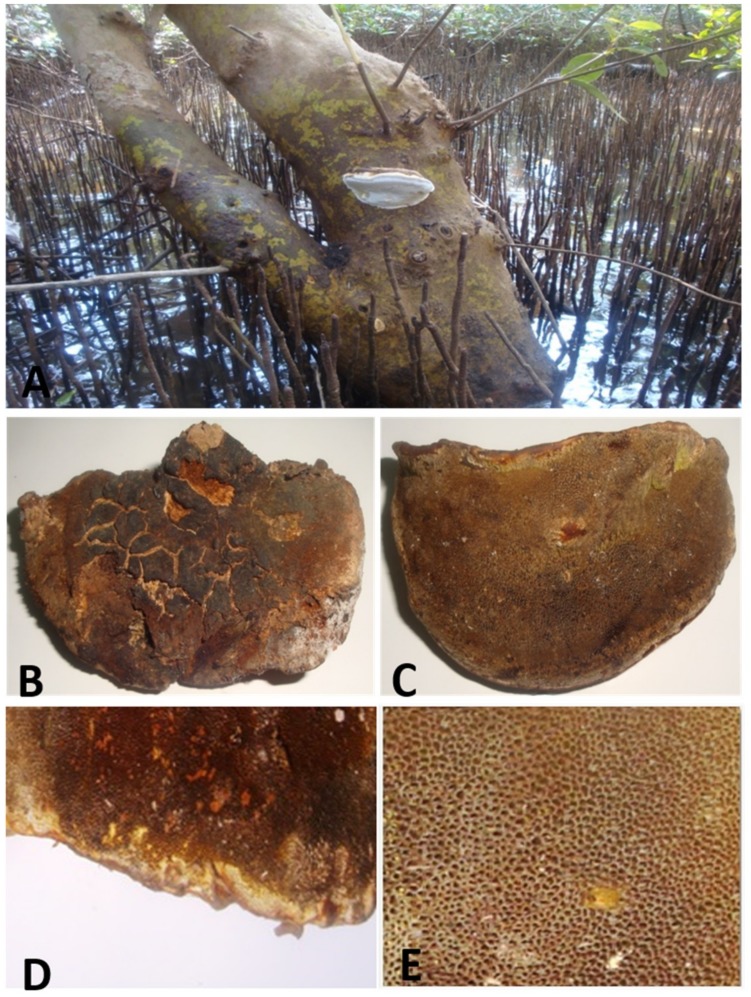
Mushroom collection and their morphological characteristics (**A**) Mushroom *Fulvifomes* sp. were spotted in *A. Marina* (**B**) Front view of fruiting body (**C**) Back view of fruiting body (**D**) Tubes (**E**) Pores.

The collected marine basidiomycetes sample from a mangrove of *Avicennia marina* (Figure 1A) was identified as *Fulvifomes* sp. and the following morphological characteristics were observed. Cap was soft basidiocarp, annual, pileate, predominantly broadly attached with tree, semicircular, solitary, convex to flat, 3.4 cm broad, 8.9 cm long, 3.7 cm thick, concentrically trench, margin thin and brown in color (Figure1B). Tubes were light brown (Figure 1D). Pores were 8–9 pores per cm, spherical to angular, brown (Figure 1E). The stalk was absent, and the flesh was hard, woody, and dark brown. The position of the fruiting body was attached to the base of mangrove *A. marina*.

### 3.2. Molecular Taxonomy of Marine Basidiomycetes

**Figure 2:**
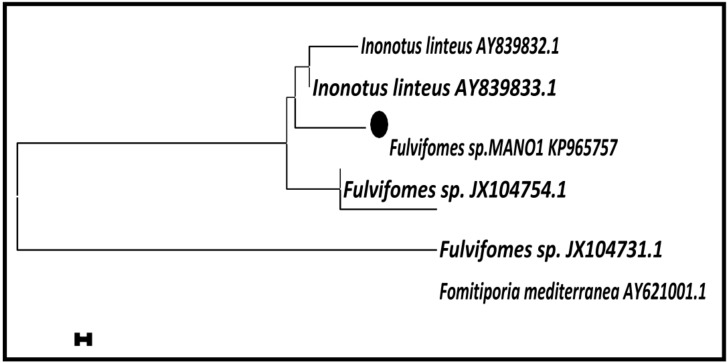
Evolutionary relationships of *Fulvifomes* sp. (MANO1 KP965757) using neighbor-joining method.

The sequenced ITS region of *Fulvifomes* sp. was deposited in GenBank under the accession No. KP965757. A BLAST search was used to assess similarities among *Fulvifomes* sp. The BLAST search confirmed the interested isolates with 97% similarity and assigned species name as *Fulvifomes* sp. This result confirmed that the isolated strains was *Fulvifomes* sp. (KP965757), which matched with *Fulvifomes* sp. (JX104754.1) and *Fulvifomes* sp., (JX104731.1) from the Genbank, and the similarities were found to be 97% and 95%, respectively (Figure 2).

### 3.3. High Performance Liquid Chromatography (HPLC) Analysis of Bioactive Compound

**Figure 3:**
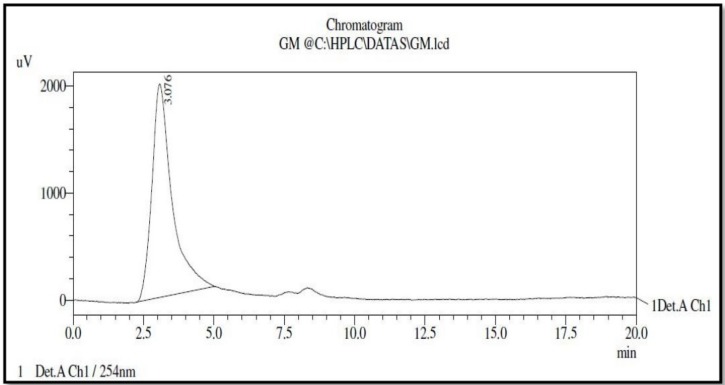
HPLC analysis of purified compound from *Fulvifomes* sp.

The result of an HPLC analysis shows that the peaks strike at twice the retention time of 1.961(Area = 92.77%) and 3.076 (Area = 7.22%). On the basis of the high abundance area (76.56%) with a retention time of 3.076, it was confirmed that the extracted compound had 90 % purity (Figure 3).

### 3.4. Infrared (IR) Spectrometry

The IR spectrum ranges exhibited the hydroxyl stretching vibration absorbance at 3398 cm^−1^. The band at 2922 and 2854 cm^−1^ is due to C–H stretching vibration. The C = C stretching and in-plane bending vibrations appeared at 1458 and 952 cm^−1^. The band at 1085 cm^-1^ is due to C–O stretching vibration. The peak at 849 and 702 cm^−1^ are due to in- and out-plane bending vibrations of the extracted compound (ergosterol peroxide).

### 3.5. Structural Prediction of Bioactive Compounds

The ^1^H NMR spectrum is depicted (500 MHz, CDCl_3_, δ, (ppm)) and results are indicated with two downfield doublet signals [δ 6.50 (H7) and 6.24 (H6)] which are the occurrence of a double bond in the skeleton of sterol (Table 1). The chemical shifts of δ 6.24 (H6) and 6.50 (H7) are indicative of a 5α, 8α-epidioxy orientation, as normally encountered at approximately 6.27 (H6) and 6.51 (H7). The assignment of the double bond in the side chain was assumed by characteristic signal at 5.16 (H22, H23), representing a double bond in the sterol side chain, with one proton attached to a hydroxyl oxygen-bearing carbon at the C-3 position which had a peak at 3.95 (H3). Six methyl group protons were observed at 18, 19, 21, 26, 27, and 28 atom positions of 0.88, 0.90, 0.99, 0.88, 0.88, and 1.21, respectively.

The ^13^C NMR (100 MHz, CDCl_3_, δ, (ppm) spectrum instigate the existence of olefins disubstituted [135.41 (C-7), 135.19 (C-23), 132.34 (C-22) and 132.36 (C-6)], represent the ergosterol derivative holding the sterol fragment. Two oxygenated quaternary carbons of 79.40 (C-5) and 82.13 (C-8) are strong indications for the structure of peroxide. Another upfield signal was observed at δ 66.45 (C-3). This is a result of the presence of the hydroxyl group at this position. The significant signals arising between the C-5 and C-8 position indicate the presence of a peroxide bridge in an inferior field. C-3 was present in the high field due to the attachment of the OH group in C-3 position. The proton and carbon NMR data suggest that the isolated compounds are ergosterol peroxide.

### 3.6. MTT Assay

**Figure 4:**
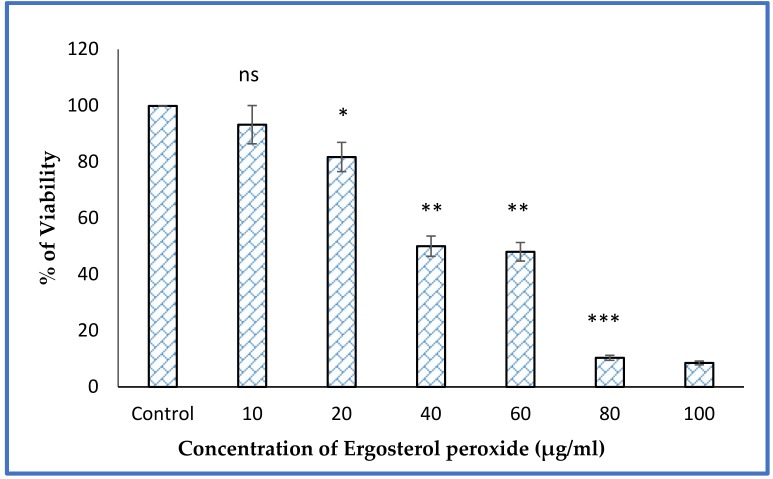
MTT assay of EP at different concentrations against MCF-7 cell line. Experiments were performed in triplicates. The results are expressed as mean ± SD. Significant difference between treated and control group were given as *(*p* < 0.05), ** (*p* < 0.01), *** (*p* < 0.001) and ns: Non-significant.

The proliferation activity of MCF-7 cells were treated with ergosterol peroxide (EP) in different concentrations, including 10, 20, 40, 60, 80, and 100 µg/mL, as determined by MTT assay. EP showed cytotoxicity on dose depended manner. The EP induced 50% of the cell (IC_50_) at a concentration of 40 µg/mL. Cell death was induced as the concentration of EP increased (Figure 4). The compound induced 90% cell death (IC_90_) at a concentration of 80 µg/mL of EP. Hence, 40 and 80 µg/mL concentrations of EP were selected for additional experiments.

### 3.7. Marine Basidiomycetes-Derived Ergosterol Peroxide (EP) Induces Reactive Oxygen Species (ROS)-Mediated Cell Death

**Figure 5:**
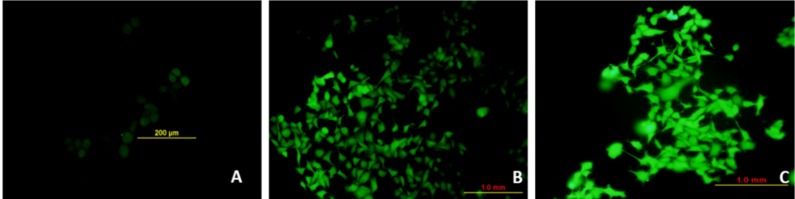
Effect of EP on production of ROS in MCF-7 cell lines. (**A**) Control (**B**) Treated with EP (40 µg/mL), (**C**) Treated with EP (80 μg/mL). ROS is an important parameter and it can be capable of producing free radicals and induced severe cell death compared to normal cells. Intracellular ROS levels were detected by fluorescent H_2_DCF-DA dye. The bright fluorescence in B and C indicating the production of ROS by inducing stress to the treated cells.

The assessment of ROS following treatment with marine basidiomycetes-derived ergosterol peroxide (EP) was determined by a DCF (2′,7′-dichlorofluorescein) fluorescent indicator. The production of ROS on MCF-7 cells was visualized under a fluorescent microscope (Figure 5). As expected, after treatment of marine basidiomycetes-derived EP, the level of ROS production was significantly increased in a dose-dependent manner (40 and 80 µg/mL of EP), which was indicated by the increased amount of fluorescent intensity (Figure 5). This result suggested that marine basidiomycetes-derived EP induces ROS-mediated apoptotic cell death. This indicates that EP was able to generate ROS and induce apoptosis in the cancer cell.

### 3.8. Et/Br Staining

**Figure 6:**
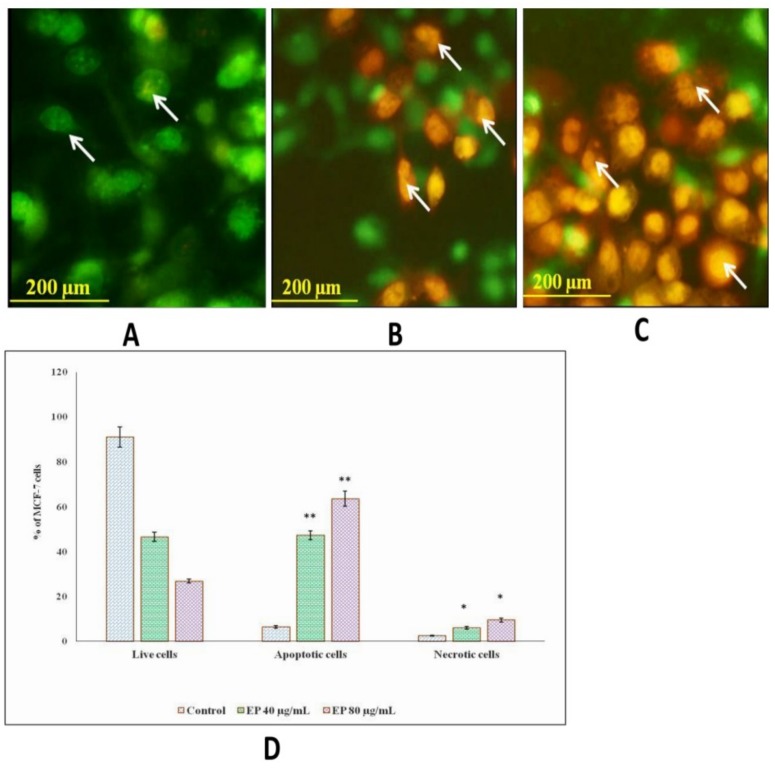
Effect of EP on apoptotic morphological changes in microscopic observation of MCF-7 cell line. The cells were treated with AO/EtBr and visualized under fluorescent microscope. (**A**) Control cells, (**B**) Treated with EP (40 µg/mL), (**C**) Treated with EP (80 µg/mL, (**D**) Experiments were performed in triplicates. The results are expressed as mean ± SD. Significant difference between treated and control group given as *(*p* < 0.05) ** (*p <* 0.01).

Ethidium bromide/acridine orange (1:1) was used to stain the marine basidiomycetes-derived ergosterol peroxide (EP) treated with MCF-7 cells after 24 h of treatment. Early apoptosis cells appeared in greenish yellow; cells stained in orange indicated late apoptosis, and necrotic cells were stained in red. In comparison with the control cells, the highest concentration, 80 µg/mL of EP treatment, extensively induced apoptosis with 63.65% and necrosis with 9.53% (Figure 6D). In addition, apoptotic cells stained with AO/EtBr exhibited nuclear fragmentation, chromatin condensation, cytoskeletal breakdown, and plasma membrane blebbing (Figure 6).

### 3.9. Loss of Mitochondrial Membrane Potential

**Figure 7:**
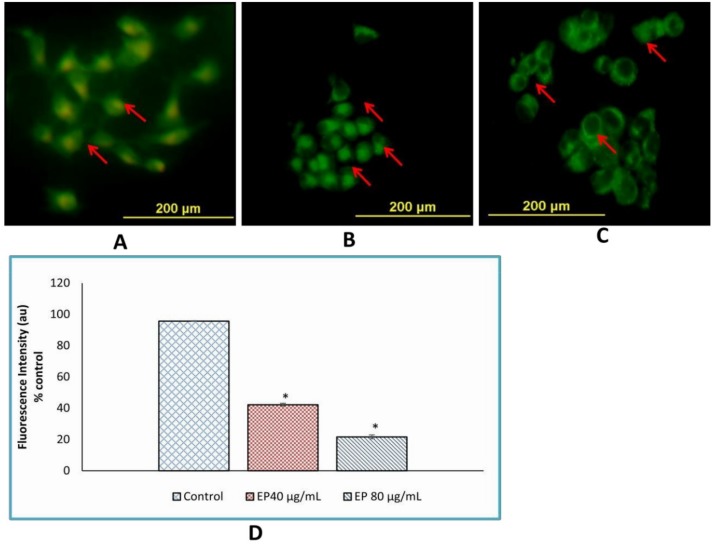
Effect of EP on mitochondrial membrane potential of MCF-7 cells. The cells were treated with (**A**) Control (untreated), (**B**) EP (40 µg/mL), (**C**) EP (80 µg/mL) for 24 h. After washing with PBS, incubated with rhodamine-123 dye (10 μg/mL) for 30 min. (**D**) Percentage of cells showing fluorescence intensity after treatment with *Fulvifomes* sp. EP on MCF-7 cells. The percentage of cells that emit only green fluorescence indicates the depolarized mitochondrial membrane loss of membrane potential and orange-red fluorescence indicates polarized mitochondrial membrane. The values are represented as mean ± SD and three independent experiments were performed. * represents significant difference compared with control (*p* < 0.05).

Loss of mitochondrial membrane potential (Δψm) with Rh-123 was studied using mitochondrial confocal microscopy. After 24 h of treatment with marine basidiomycetes-derived ergosterol peroxide (EP), membrane loss was promoted in MCF-7 cells, dependent on concentration. The green fluorescence of treated cells indicated that marine basidiomycetes-derived EP induced apoptosis through the loss of (Δψm) (Figure 7). In contrast, untreated (control) cells appeared healthy and with high mitochondrial membrane potential. The orange-red fluorescent intensity was found to be increased in the control in comparison with the treated cells. As expected, nearly 75% of the fluorescent intensity was diminished in treated groups. The concentration of 40 μg/mL and 80 μg/mL of EP treated wells showed significant loss of Δψm with *p* < 0.05 (Figure 7D).

### 3.10. Hoechst Staining 33342

**Figure 8:**
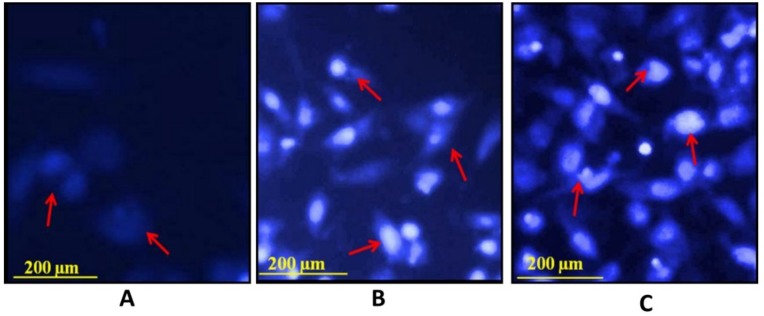
Effect of EP on nuclear damages in MCF-7 cells were observed with Hoechst 33258 staining under fluorescence microscopy. (**A**) Control (untreated), (**B**) EP (40 µg/mL) and (**C**) EP (80 µg/mL) for 24 h.

The assessment of apoptosis in breast cancer cells by marine basidiomycetes-derived ergosterol peroxide (EP) was performed by Hoechst staining 33342. Treatment with 40, 80 µg/mL of EP for 24 h in MCF-7 cells produced intense, positive staining for condensed nuclei and was indicative of apoptosis. Untreated control cells appeared in blue-nuclei; however, MCF-7 cells treated with 40, 80 µg/mL (Figure 8) of EP were absorbed in bright blue-nucleus, indicating chromatin condensation. As expected, the highest dose of 80 µg/mL induced highest fluorescence intensity in comparison with the lower dose of 40 µg/mL.

## 4. Discussion

In the present study, a mushroom collected from the mangrove of *Avicennia marina* was identified as *Fulvifomes* sp. A previous study reported that members of the *Fulvifomes* species are unique in their morphological characteristics, as evidenced by those found on the mangrove tree at the Ranong Biosphere Reserve, located on the west coast of the Thai Peninsula [12]. The DNA sequences obtained in this study were deposited in the GenBank database with accession number KP965757. An ITS sequence of *Fulvifomes* was subjected in the BLAST program to generate the significant alignment and closely matched the query sequence. The ITS sequence was isolated from the pure culture of *Fulvifomes* and showed 97% similarity to *Fulvifomes* sp. (JX104754.1) and 96% similarity to *Fulvifomes* sp. (JX104757.1), an isolate from the mangrove tree in Thailand [13]. The aim of the current study is the isolation of apoptosis-inducing compounds from the marine basidiomycetes. This is the first report to examine the marine basidiomycetes mushroom. Bioactive compounds extracted from marine basidiomycetes have significant pharmaceutical applications. In this study, EP was extracted from the *Fulvifomes* sp. Similarly, the EP derived from the entomopathogenic fungi was studied for bioactive potential [14,15].

EP from the mangrove-dwelling mushroom *Fulvifomes* sp. induced the inhibition of breast cancer cell lines MCF-7 growth. Another study has confirmed that medicinal, mushroom-derived EP exhibited promising antitumor activity through β-catenin and STAT3 pathways in ovarian cancer cells [16]. EP derived from neungyi mushrooms (*Sarcodon aspratus*) induced a strong cytotoxic effect against prostate cancer cells lines of DU 145 and M2182 [17]. Depending on the concentration of EP, the compounds induced 50% cell death at a concentration of (IC_50_) 40 µg/mL compared to the lower concentration of 20 µg/mL. The drug exhibited cytotoxicity in a dose-dependent manner. Cell death was induced as the concentration of EP increased. The compound killed 90% of cells (IC_90_**)** at a concentration of 80 µg/mL of EP. Similarly, *Ganoderma lucidum*-derived EP induced 50% cell death at the inhibitory concentration of (IC_50_) 40 µg/mL and 90% cell death at (IC_90_) 100 µg/mL [18]. Numerous studies have shown the therapeutic potential of EP. Subsequently, EP isolated from the acetone extract *S. aspratus* induced the apoptosis after 24 h in HL60 leukemia cells [19]. An earlier study reported that EP can potentially induce the antitumor effects in multiple myeloma U266 cells; simultaneously, it can act as an antiangiogenic agent through the targeting of the JAK2/STAT3 signaling pathway [20]. Chaga mushroom (*Inonotus Obliquus*) derived EP inhibited the growth of colorectal cancer cell (CRC) lines which resulted in apoptosis induction, confirmed by FACS (Fluorescence-activated cell sorting) analysis [21]. The EP attenuated the prostate cancer cell by triggering an apoptotic process [22].

Reactive oxygen species (ROS) induces oxidative modification in the DNA of other small cellular molecules. ROS level was assessed after 24 h of incubation with EP. Changes were compared with untreated control wells and interpreted as either an increase or decrease of the amount of internal ROS. Treatment with EP caused rapid increases of intracellular ROS in MCF-7 cells. The present study noticed that a significant increase in ROS levels was reached at 80 µg/mL of EP-treated cells. Previous studies have reported that EP directly generates the intracellular ROS or through an alteration of the redox state. Accordingly, it upregulates the oxidative, stress-sensitive responsible genes and downregulates the STAT1 and interferon-inducible genes by increasing the intracellular levels of ROS [23]. Similarly, the anticancer activity of EP is in good agreement with previously published findings on the inhibition of cancer cell proliferation [24].

## 5. Conclusions

The present study successfully isolated EP (ergosterol peroxide) from marine basidiomycetes. The extracted ergosterol peroxide from *Fulvifomes* sp. can induce apoptosis in breast cancer cells. Accordingly, EP could be a promising candidate for breast cancer therapy via apoptosis induction. Although in vitro studies confirm the anticancer property of EP, further in vivo studies are necessary to analyse its potential use as an anticancer drug in the pharmaceutical and biomedical industries.

## Figures and Tables

**Table 1:** ^1^H and ^13^C NMR spectral data for ergosterol peroxide.

Atom Position	δ^13^C (ppm)	δ^1^H(ppm)
1	36.96	2.1
2	30.14	1.97
3	66.45	3.95
4	51.17	0
5	79.4	5.24
6	132.36	6.24
7	135.41	6.5
8	82.13	5.23
9	34.72	0
10	36.96	0
11	20.86	0
12	39.38	0
13	44.57	0
14	51.71	0
15	28.58	0
16	23.4	0
17	56.26	1.21
18	12.86	0.88
19	18.14	0.9
20	39.64	0
21	28.86	0.99
22	132.34	5.16
23	135.19	5.16
24	42.78	1.85
25	33.06	1.47
26	19.06	0.88
27	19.61	0.88
28	17.53	1.21
